# Active Packaging-Releasing System with *Foeniculum vulgare* Essential Oil for the Quality Preservation of Ready-to-Cook (RTC) Globe Artichoke Slices

**DOI:** 10.3390/foods10030517

**Published:** 2021-03-02

**Authors:** Valeria Rizzo, Sara Lombardo, Gaetano Pandino, Riccardo N. Barbagallo, Agata Mazzaglia, Cristina Restuccia, Giovanni Mauromicale, Giuseppe Muratore

**Affiliations:** Di3A, Dipartimento di Agricoltura, Alimentazione e Ambiente, University of Catania, via S. Sofia 100, 95123 Catania, Italy; vrizzo@unict.it (V.R.); sara.lombardo@unict.it (S.L.); g.pandino@unict.it (G.P.); rbarbaga@unict.it (R.N.B.); agata.mazzaglia@unict.it (A.M.); g.mauromicale@unict.it (G.M.); g.muratore@unict.it (G.M.)

**Keywords:** essential oil emitter, globe artichoke genotype, quality parameters, microbial growth, antioxidants’ retention

## Abstract

Two globe artichoke genotypes, “Spinoso sardo” and “Opera F1”, have been processed as ready-to-cook (RTC) slices and refrigerated at 4 °C for 12 days (i) to evaluate the suitability to be processed as RTC slices; (ii) to evaluate the effect of a *Foeniculum vulgare* essential oil (EO) emitter, within an active package system, to delay quality decay, thus extending shelf life; (iii) to estimate the impact of EO emitter on the sensory profile of the RTC slices after cooking. Results revealed that both globe artichoke genotypes possess a good attitude to be processed as RTC product. “Opera F1” showed the best performances for color parameters, texture and chemical indexes, while “Spinoso sardo” showed lower mass loss (ML) over the storage time. The addition of EO emitter slowed down the consumption of O_2_, better preserved texture when compared to the control and more effectively control polyphenol oxidase (PPO) activity and antioxidants’ retention during the cold storage. Microbial counts in control globe artichoke RTC slices were significantly higher than those packed with EO emitter, confirming the inhibiting role played by EO of *F. vulgare*. In addition, the EO emitter did not influence negatively the sensory profile of RTC globe artichoke slices after microwave cooking.

## 1. Introduction

Food market demand is increasingly focused on food products with a high level of healthy compounds. In this context, globe artichoke (*Cynara cardunculus* L. var. *scolymus* (L.) Fiori = *C*. *scolymus* L.), whose production for a long time was limited to the native Mediterranean region, is becoming increasingly popular and desired by consumers all over the world for its taste and functional properties [1,2,3]. The edible immature inflorescence (named as capitulum or head), which is composed of a receptacle surrounded by thickened bracts, is destined for fresh consumption or industrially processed production in several ways [4]. Particularly, the complex trimming operations necessary for heads to be freshly consumed, especially for the genotypes with spines, have increased recently the industrial production of ready-to-cook (RTC) globe artichoke slices [5,6], which can definitively balance consumer’s demand due to easier use and freshness and better quality of them.

A good opportunity to improve sales for food industry is the production of fresh-cut vegetables; minimally processed products are one of the rapid growing sectors in the food industry due to the convenience and nutritional value, but it is well known that processing operations, mainly cutting, have the disadvantage of accelerating enzymatic browning phenomena and microbial spoilage, respect to the whole vegetables, due to the loss of compartmentalization of vegetable cells. Specifically, the enzymatic browning is mainly attributed to the polyphenol oxidase (PPO; EC 1.14.18.1), which generates dark pigments called melanoidins, unacceptable in terms of safety, since they could support the microbial growth. Additionally, although moderate, stress conditions or short storage times could be due to the loss of sensory and nutritional quality of the processed globe artichokes [5,7,8,9,10,11]. To slow down such quality decrease, the use of natural antimicrobials, such as essential oils (EOs), instead of chemical agents has emerged as a promising strategy [12]. Active packaging systems have been successfully applied to minimally processed vegetables [13]. Among them, active packaging releasing systems (emitters) are applied to add compounds into the headspace to improve shelf life of food, like antimicrobial substances, CO_2_, antioxidants, or ethanol. Plants are relevant sources of bioactive molecules possessing antimicrobial activities against pathogens and spoilage microorganisms. The different compounds having antimicrobial activities in plant are alkaloids, phenolics, terpenes, terpenoids, flavonoids, essential oil, etc. [14,15]. The positive effects of the addition of EOs into individual carrier, as emitters inside packaging, instead of the incorporation into polymers, is to avoid any undesired change of properties of the polymeric films [16].

An important role in plant defense is played by EOs, secondary metabolites, some of which exert powerful antimicrobial activities [12,17,18], are classified as Generally Recognized As Safe (GRAS) [19], and are often used for the inhibition of pathogenic bacteria in foods.

EOs have been deeply studied also as active components in bio-based emulsified films and coatings. The EO from *Foeniculum vulgare* seeds, which is rich in trans-anethole, has been demonstrated to possess antioxidant activities [16], good antimicrobial activity against food-borne pathogens such as *Shigella dysenteriae* [20], *Escherichia coli* [21], and other pathogenic bacterial and fungal species [22,23,24]. With reference to food packaging application, recently Rizzo et al. [11] demonstrated the notable efficacy of the locust bean gum (LBG) coating with the addition of *F. vulgare* EO in preserving quality of RTC globe artichoke slices of “Spinoso sardo” during refrigerated storage for 11 days. However, although EO-based packaging has the ability to increase food shelf life, the selection of EO-based materials should be done on the basis of the compatibility with foods in terms of flavor to avoid any negative impact of EOs on the sensory profile.

The aims of this study, performed on RTC globe artichoke slices stored at 4 °C for 12 days, were therefore: (i) To evaluate the suitability of two globe artichoke genotypes, “Spinoso sardo” and “Opera F1”, to be processed as RTC slices; (ii) to evaluate the effect of a *F. vulgare* EO emitter, within an active package system, as a strategy to slow down quality decay, and thus increasing shelf life; (iii) to estimate the impact of EO emitter within the package on the sensory profile of the RTC slices after microwave cooking.

## 2. Materials and Methods

### 2.1. Experimental Field, Plant Material, and Management Practices

Experimental trial was performed during 2014–2015 on Siracusa plain (36°58′ N, 15°11′ E; 53 m a.s.l. (metres above sea level)), one of the main Italian site for globe artichoke crop. The soil, classified as calcixerollic xerochrepts [25], had the following characteristics: pH 7.7, 50% sand, 18% silt, 32% clay, 6% limestone, 1.9% organic matter, 0.18% total N, and 30 mg kg^−1^ of available P_2_O_5_ and 280 mg kg^−1^ of exchangeable K_2_O. The climate of the area is semiarid-Mediterranean, with mean long-term monthly maximum and minimum temperatures from 14.8 (January) to 30.6 °C (July) and from 7.8 (January) to 22.3 °C (August), respectively [26].

“Spinoso sardo”, an early reflowering multiclone globe artichoke genotype, with conical green heads characterized by purple shades and big yellow spines [27], and “Opera F1”, a hybrid producing purple-colored spherical heads, were studied. Planting was performed by either semi-dormant offshoots or seedling) in August 2014, by adopting a randomized block experimental design with 4 replications and a planting density of 1.0 plant m^−2^. Crop management was carried out in agreement with the usual commercial practice of the cultivation area.

### 2.2. Head Harvest, Post-Harvest Treatments, and Sampling

Around 100 globe artichoke heads per each plot were harvested at the beginning of March at the marketable stage [28], transported to the Department of Agriculture, Food and Environment, University of Catania (Di3A) laboratories under controlled temperature and processed as a RTC product the day after. Samples were treated as reported by Licciardello et al. [9]. Briefly, all the inedible parts and the heads” tips were first eliminated and then heads were cut into 5 mm thick slices by using a manual cutting machine, sanitized and treated in anti-browning solution as reported by Rizzo et al. [11]. Slices were dried in a manual centrifuge, to eliminate residual water solution and then were placed in PET trays (16 × 11 × 3.5 cm), filled them up to 100 ± 10 g for each tray and packaged into a semi-permeable polyolefine film (SPP) (SP/BY 19 micron-System Packaging s.r.l., Siracusa, Italy) having an oxygen transpiration rate (OTR) of 3000 cm^3^/m^2^/24 h at 23 °C and 0% relative humidity (RH). Totally 80 trays were filled with “Opera F1” and “Spinoso sardo” slices respectively. Inside a half of the totally package for each genotype (40) was placed a small square (1 × 1 cm) of sterilized TNT textile, soaked with 150 µL of *F. vulgare* EO (0.75% *w*/*w*) produced by Rao Erbe (Catania, Italy) immediately before the hermetical sealing of trays with SPP plastic bags (20 × 15 cm) by a sealing bar (Lafayette Model SK-410, Frosinone, Italy). Samples were kept at 4 ± 0.5 °C and 90–95% RH up to 12 days and analysis were done at the processing day (T0), and after 5, 8 and 12 days of refrigerated storage (Figure S1).

### 2.3. Weight Loss and Headspace Gas Composition

Every package was marked with the starting weight. Then, at each sampling time three different trays for each post-harvest treatment were chosen (5, 8 and 12 day) and immediately weighed before further analysis. Mass loss (ML) was expressed as % of the initial sample weight at T0. The headspace gas composition, to quantify carbon dioxide and oxygen, was measured using a CheckPoint portable gas analyzer (MOCON Europe A/S (Dansensor), Ringsted, Denmark). Analysis were done on three replicates every sampling time.

### 2.4. Color Analysis

Surface color of RTC globe artichoke slices was assessed according to CIE L*a*b* scale as stated by Rizzo et al. [29], using a portable colorimeter (NR-3000, Nippon Denshoku Ind. Co., Ltd., Tokyo, Japan), correctly calibrated, and illuminant D65/10°. Data were showed as L* = lightness, a* = redness, b* = yellowness and calculated as ΔE* = (ΔL*^2^ + Δa*^2^ + Δb*^2^)^1/2^ pointing at the total color difference, to better assess the overall color changes during storage as previously done by Licciardello et al. [30].

### 2.5. Texture Analysis

The texture was measured by applying a 500 N nominal force cell and a stainless-steel probe (length 5 mm) and measuring the maximum shear force, using instruments and data elaboration as reported by Rizzo et al. [11] Results were the average of 6 measurements.

### 2.6. Microbiological Analyses

Microbiological determinations were performed on 25 g of RTC artichokes, aseptically sampled from each package and homogenized in a Lab-Blender 400 (Brinkmann, Westbury, NY, USA) for 3 min with 225 mL of Ringer solution (Oxoid, BR0052, Basingstoke, UK). The determined microorganisms were: Total aerobic mesophilic bacteria (MB) and total aerobic psychrotrophic bacteria (PB) on plate count agar (PCA, Oxoid, CM325, Basingstoke, UK) with cycloheximide 0.1% solution (Oxoid, SR0222, Basingstoke, UK), incubated at 30 °C for 48 h and at 7 °C for 5–10 days, respectively; at; yeasts and molds (YM) on Sabouraud Dextrose Agar (SDA, Oxoid, CM0041, Basingstoke, UK) supplemented with 0.1 g L^−1^ chloramphenicol (Oxoid, SR0078, Basingstoke, UK), incubated at 25 °C for 48–72 h; Enterobacteria (TEB) on violet red bile glucose agar (VRBGA, Oxoid, CM0485, Basingstoke, UK), incubated at 37 °C for 24 h; *Escherichia coli* on brilliance E. coli selective agar (Oxoid, CM1046, Basingstoke, UK), incubated at 37 °C for 24 h; *Pseudomonas* spp. on Pseudomonas Agar Base (CM0559, Oxoid, Basingstoke, UK), supplemented with Pseudomonas CFC selective agar supplement (SR0103, Oxoid, Basingstoke, UK) and incubated at 25 °C for 48 h. All microbiological analyses were performed in triplicate and expressed as average log10 CFU g^−1^.

### 2.7. Chemical and Enzymatic Analyses

An amount of RTC globe artichoke slices from each package was freeze dried (Christ freeze drier; Christ, Osterode am Harz, Germany) for performing the following chemical determinations.

L-ascorbic acid content (AsAC) was evaluated following the HPLC method proposed by Lombardo et al. [5] and expressed as mg kg^−1^ of DM (dry matter).

A modified Folin-Ciocalteu method [4] was adopted for the determination of the total polyphenol content (TPC), which was expressed as g kg^−1^ of DM, using chlorogenic acid as a standard; in the same extracts used for TPC analysis, the antioxidant activity (AA), expressed in terms of DPPH (2,2-diphenyl-1-picrylhydrazyl) percentage of inhibition, was determined using the method reported by Brand-Williams et al. [31] and calculating the results as follows:

(1) AA =(AC0−AS30)÷AC0 × 100

where AC0 is the absorbance of the control assay (no extract) and AS30 the absorbance of the sample after 30 min.

The determination of PPO activity was spectrophotometrically performed [32,33], using catechol as a phenolic substrate. defining one unit of PPO activity as the level of enzyme able to increase the absorbance by 0.001 min^−1^.

All these chemical determinations were conducted by using bi-distilled water and analytical or HPLC grade reagents and solvents obtained from Sigma-Aldrich (Milan, Italy).

### 2.8. Sensory Analyses

The UNI EN ISO 13,299 [34] method was adopted to evaluate changes in sensory characteristics of samples. Ten judges (six female and four male, 24–40 years old and a plurennial expertise in the sensory evaluation of vegetables) were selected from the Di3A and were trained as indicated by ISO 8586 [35] in four meetings using different kind of globe artichoke samples, to achieve a common language for the description of sensory traits and to familiarize themselves with scales and procedures.

According to Rizzo et al. [11,17] the following main sensory attributes were considered: One for appearance (freshness), six for odor (herbaceous, globe artichoke, fennel, apple, potato, and off-odor), six for flavor (herbaceous, globe artichoke, fennel, apple, potato, and off-flavor), two for taste (sweet and bitter), one for tactile (astringent), and overall score. Before the sensory evaluation, the samples were cooked using commercially available microwave cooking bags (FRIO; Sphere France S.A.S., Paris, France) in a microwave oven (Whirlpool, Benton Harbor, MI, USA) at 700 W for 10 min.

The panel evaluated the randomized samples adopting a scale from 1 (absence of sensation) to 9 (extremely intense) in the sensory laboratory [36] of the Di3A. Water was provided for mouth rinsing between tests. Computerized data-collection software was used (FIZZ, Software Solutions for Sensory Analysis and Consumer Tests, Biosystemes, Couternon, France).

### 2.9. Statistical Analysis

Bartlett’s test was adopted to verify the homoscedasticity, and then the data (when necessary transformed by Bliss transformation prior to statistical analysis) were subjected to a three-way analysis of variance (ANOVA) as a factorial combination of “genotype (2) × postharvest treatment (2) × storage time (4)”. For each sensory attribute a two-way ANOVA as a factorial combination of “genotype (2) × postharvest treatment (2)” was performed. Means were separated by LSD test, when the *F*-test was significant (*p* ≤ 0.05). Limitedly to AsAC, TPC, and AA, the correlation analysis was carried out to estimate their relationship. The software package Statgraphics^®^ CenturionXVI (Statpoint Technologies, INC., The Plains, VA, USA) was used to perform ANOVA.

## 3. Results and Discussion

### 3.1. Fresh Weight Loss, Headspace Concentration, Color, and Texture

It is assumed that fresh globe artichoke heads are cooked prior to consumption. The USDA National Nutrient Database for Standard Reference reports that about 40% of a fresh head is edible, while the USDA Food and Nutrient Database for Dietary Studies indicates that about 6% of this edible share is further lost through cooking. Therefore, while the consumers have to consider only the weight lost through cooking of RTC globe artichoke slices, producers have to consider also the whole mass loss to define their profit. As previously reported by Licciardello et al. [9], the most important function of the packaging is to decrease the fresh vegetable moisture loss by respiration and surface evaporation mechanisms, thus increasing product shelf life. For this reason, the SPP film was selected for our study, considering the positive performance in reducing fresh weight loss under refrigerated storage conditions at a maximum−1%, due to its low water vapor transmission rates [6]. The ANOVA for the ML highlighted a significant influence of the studied main effects (Genotype (G), Treatment (T) and Storage time (St)), as well as a high statistical significance for the interaction “G × St”, “T × St” and “G × T × St” (Table 1). As expected, ML increased during the refrigerated storage (Table 2), reaching its maximum value around 2%, which resulted quite higher than the observed values for other globe artichoke genotypes previously studied (i.e., “Apollo”, “Exploter”, and “Spiosodi Palermo”) [9].

**Figure 1:**
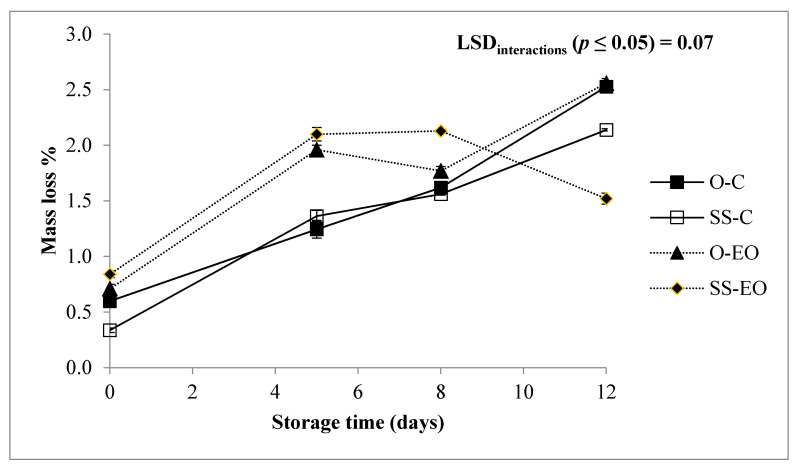
Mass loss (%) of RTC globe artichoke slices as affected by “genotype × post-harvest treatment × storage time” interaction. O: “Opera F1”; SS: “Spinoso sardo”; C: Control; EO: Samples packed with essential oil of *Foeniculum vulgare.* Data are reported as means ± standard deviation.

ML was slightly higher in “Opera F1” at the end of the experimental trial (12 day) respect to “Spinoso sardo”, while samples with EO”s pad began with higher values that were maintained until 8 days (Figure 1). It is evident how the RTC globe artichoke slices packed with EO emitter follows the same path of the control, underlining more correspondence to the genotype then to the treatment (Figure 1).

**Figure 2:**
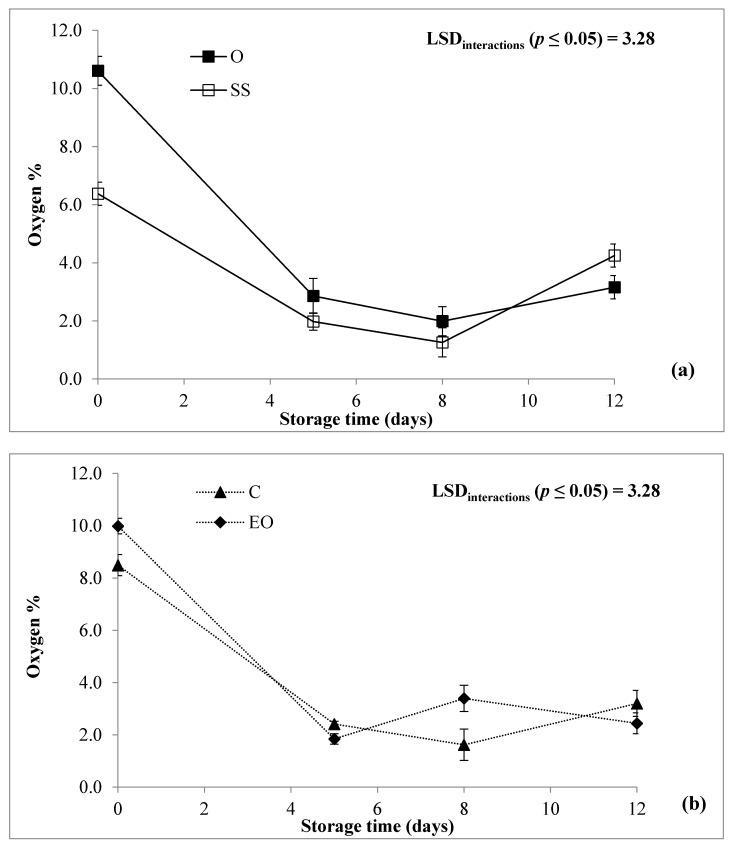
Oxygen (%) of RTC globe artichoke slices as affected by “genotype × storage time” (**a**); “post-harvest treatment × storage time” (**b**); interaction. O: “Opera F1”; SS: “Spinoso sardo”; C: control; essential oil (EO): Samples packed with essential oil of *F. vulgare*. Data are reported as means ± standard deviation.

With reference to the two components of packaging headspace, both O_2_ and CO_2_ were not influenced by G, as expected, thus confirming previous results [9], while St influenced the percentage of O_2_ and CO_2_ for *p* ≤ 0.001 and *p* ≤ 0.01, respectively (Table 1). Changes in O_2_ and CO_2_ concentration slowed down during the cold storage (Table 2), while only the O_2_ concentration varied during the storage time depending on G and T as reported in Figure 2a,b, respectively. Thus, the headspace O_2_ concentration changed rapidly during the first 5 days of storage [5,9], then reaching an equilibrium concentration until the end of the storage for both G and T. “Opera F1” displayed higher O_2_ concentration than “Spinoso sardo” only at the processing day, while both genotypes reported similar values of this parameter throughout the storage time (Figure 2a). The presence of EO emitter instead seemed to slow down the consumption of O_2_, reaching after 12 days comparable headspace gas concentrations (Figure 2b). As explained by Ghidelli et al. [37], preparing globe artichoke heads by removing inedible parts led to a sharp drop in O_2_ and an increase in CO_2_.

**Figure 3:**
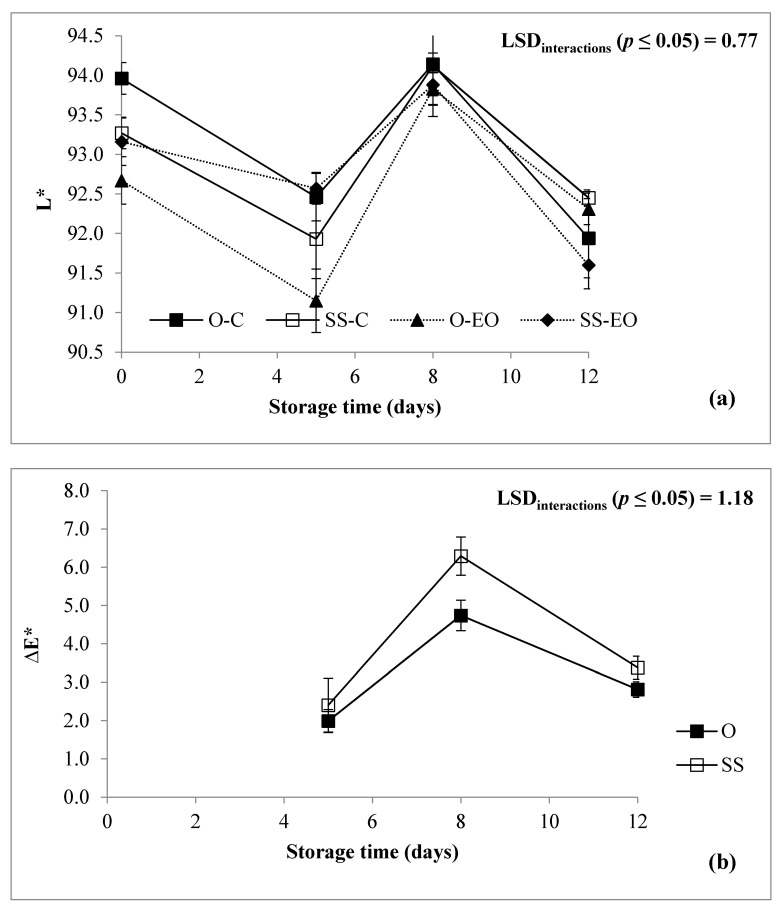

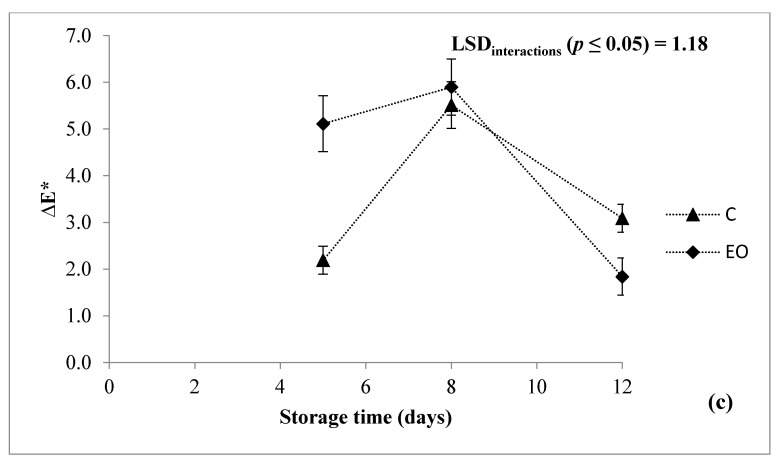
Color parameters of RTC globe artichoke slices as affected by “genotype × post-harvest treatment × storage time”(**a**) “genotype × storage time” (**b**); “post-harvest treatment × storage time”(**c**) interaction. L*: Lightness; ΔE* = total color difference; O: “Opera F1”; SS: “Spinoso sardo”; C: control; EO: Samples packed with essential oil of *F. vulgare*. Data are presented as means ± standard deviation.

In fresh cut vegetables, appearance color represents the marketability value of the product itself. Considering the studied chromatic indexes, the ANOVA showed as all the color parameters were mainly affected by St (Table 1) in agreement with previous findings, which reported as St resulted the predominant factor influencing color [10]. L* was affected by T, St and “G × T × St” interaction (Table 1; Figure 3a). L* decreased in both genotypes and post-harvest treatments during the first 5 days, as confirmed by previous study [11], then increased in the following 3 days and again slowed down at the end of the trial. “Opera F1” had higher values from T0 to the 8th day of St, to rapidly decrease at the last day of the trial respect to “Spinoso sardo”, which instead had higher values at the end of St. Such path may be explained considering that studying the color by a colorimeter, a decrease of L* can be an indication of browning appearance as well as an increase in L* can be linked with the progress of whiteness in the samples for the same reason [38].

The ∆E was characterized by “G × St” and “T × St” interactions (Table 1). In this view, the genotype “Spinoso sardo” recorded higher values than “Opera F1”, thus indicating greater changes during the storage period (Figure 3b), and the presence of EO’s pad did not improve color appearance, since the increase of ∆E after 8 days corresponds to a worse color vision by the consumer (Figure 3c), but at the end of the storage it was smaller than control. Generally, ∆E assessed color changes taking place during St, which is not simply related to browning or to other perceived aspects, but it is the expression of the complexity of different (unspecified) effects [30].

**Figure 4:**
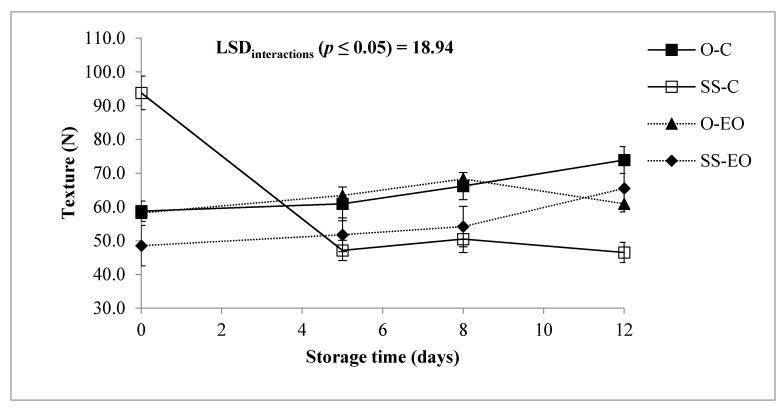
Texture (N) of RTC globe artichoke slices as affected by “genotype × post-harvest treatment × storage time” interaction. O: “Opera F1”; SS: “Spinoso sardo”; C: Control; EO: Samples packed with essential oil of *F. vulgare.* Data are showed as means ± standard deviation.

In RTC globe artichoke slices texture is doubly important because it has to satisfy the consumer at package’s opening, but also after cooking. Texture was significantly influenced by interactions “G × St”, “T × St” (both at *p* ≤ 0.05) and “G × T × St” (*p* ≤ 0.01), as reported in Table 1. Texture in “Opera F1” did not change substantially throughout the storage time, while “Spinoso sardo” reported an immediate loss of about 50% after the first 5 days, then settling on almost constant but lower values than those recorded for “Opera F1” (Figure 4). As studied in the past, texture increased in “Opera F1” for the loss of water by the vegetable, possibly derived by lignin production, while the resistance decreased in “Spinoso sardo” during storage for the liberation of proteolytic and pectolytic enzymes caused by the cellular breakdown [11]. Also, the EO treatment showed a fairly increasing trend, keeping values after 5 days of storage slightly higher than the control which showed a drop off after 5 days (Figure 4).

### 3.2. Antioxidant Compounds Content and Activity

Inhibition of browning phenomena is a main challenge in the production of RTC globe artichoke slices with the aim of ensuring a longer shelf life and more suitable visual qualitative attributes of the product [9,10]. Natural antioxidant compounds, either endogenous in globe artichoke (e.g., ascorbic acid and polyphenols) or applied as safe preservatives (e.g., EOs), are found to be efficient in preventing the qualitative damages during cold storage of minimally processed vegetables [6,30]. However, several factors influenced the levels of antioxidant compounds in globe artichoke [39,40,41]. Here, the ANOVA results revealed the significant influence of the studied main effects (G, T and St) and the “T × St” interaction for both the AsAC and TPC (Table 1).

**Figure 5:**
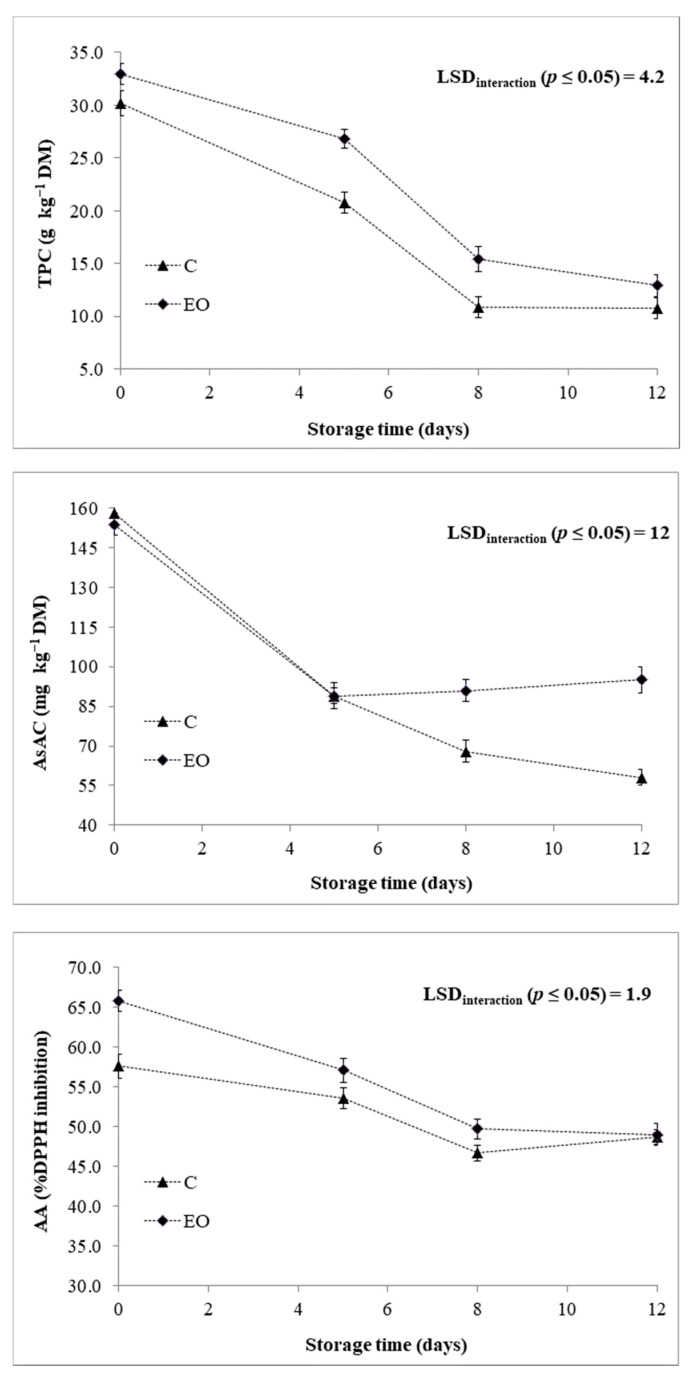
Chemical traits of RTC globe artichoke slices as affected by “post-harvest treatment × storage time” interaction. TPC: Total polyphenol content; AsAC: Ascorbic acid content; AA: Antioxidant activity; C: Control; EO: Samples packed with essential oil of *F. vulgare.* Data are presented as means ± standard deviation.

**Figure 6:**
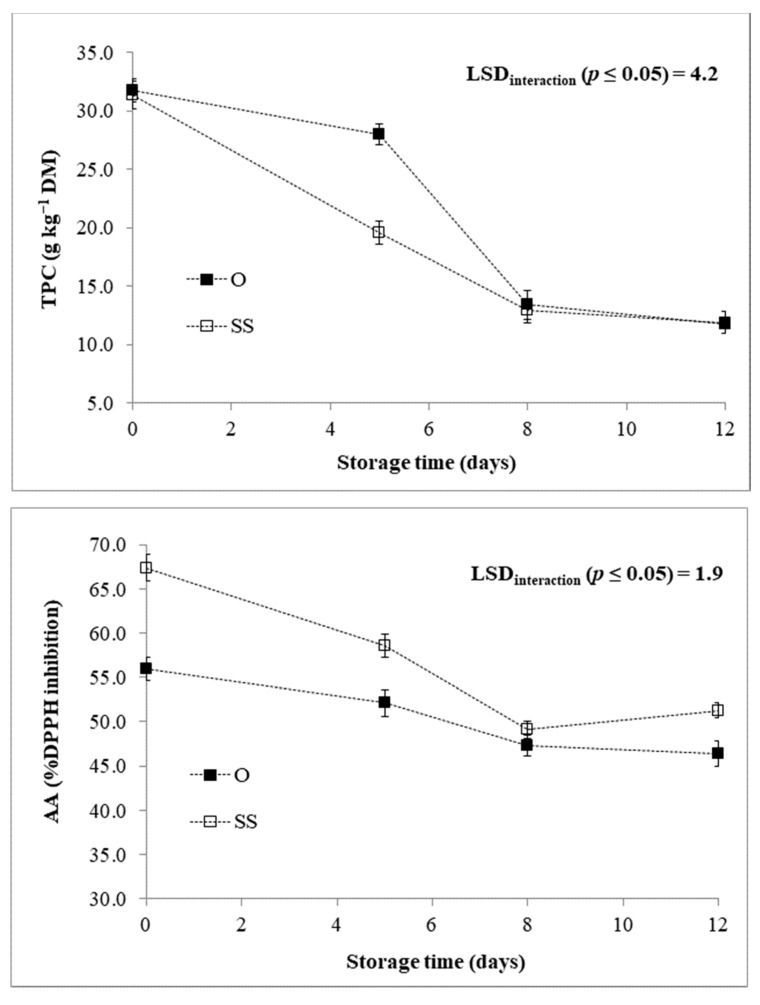
Chemical traits of RTC globe artichoke slices as affected by “genotype × storage time” interaction. TPC: Total polyphenol content; AA: Antioxidant activity; O: “Opera F1”; SS: “Spinoso sardo”. Data are presented as means ± standard deviation.

Both TPC and AsAC decreased throughout the storage time, regardless of post-harvest treatment and genotype (Table 2). For the TPC, this may depend upon the conversion of phenolic compounds to the relative quinones (scarcely reactive to the Folin–Ciocalteu method) by PPO [42]. For such reason, when the PPO activity declined, the TPC was quite stable in both post-harvest treatments (Figure 5) and genotypes (Figure 6) passing from 8 to 12 days of cold storage. Our results also indicated that TPC decreased much more slowly in presence of EO (Figure 5), which can be imputed to the additional polyphenols provided by packing RTC globe artichoke slices with EO emitter. In particular, after 5 and 8 days of cold storage, EO was able to increase the TPC than the control C (Figure 5). Similarly, during the storage time, samples presented a different AsAC retention with reference to the post-harvest treatment subjected (Figure 5). In particular, after 5 days of cold storage EO reinforced the AsAC retention as also highlighted for the TPC.

The AA (measured by DPPH assay) had a good correlation with both TPC (*r* = 0.821 ***) and AsAC (*r* = 0.775 ***). As reported previously [6,10], globe artichoke extracts possess a high and exploitable AA as safe qualitative preservative. Here, it was significantly influenced by the studied main effects (G, T, and St) and the interactions “T × St” and “G × St” (Table 1). On the whole, the AA trend is consistent with those highlighted for AsAC and TPC, decreasing throughout the storage period, although with a different extent depending on post-harvest treatment (Figure 5) and genotype (Figure 6). Indeed, at each sampling time it was higher in samples packed with EO emitter than C (Figure 5), as a result of the enhanced level of antioxidants in the product provided by EO of *F. vulgare*.

A significant influence of genotype was found for all the three traits here commented (Table 1). In this view, the decoding of globe artichoke genome may lead to the constitution of genotypes with enhanced yield and quality features [3,43,44]. Here, it is noteworthy to underline as “Opera F1” displayed higher TPC and AsAC than “Spinoso sardo”, as well as a higher AA consequently (Table 2). However, “Opera F1” experienced a higher reduction of AA at each sampling time (Figure 6), which could be related to its polyphenolic profile. Indeed, polyphenols are recognized as the main contributors to the AA of globe artichoke extracts [1,10].

### 3.3. Polyphenol Oxidase (PPO) Activity

The ANOVA revealed that the three sources of variation (G, T and S) were all individually significant for the PPO parameter, but only “G × St” and “T × St” interactions were significant (Table 1).

**Figure 7:**
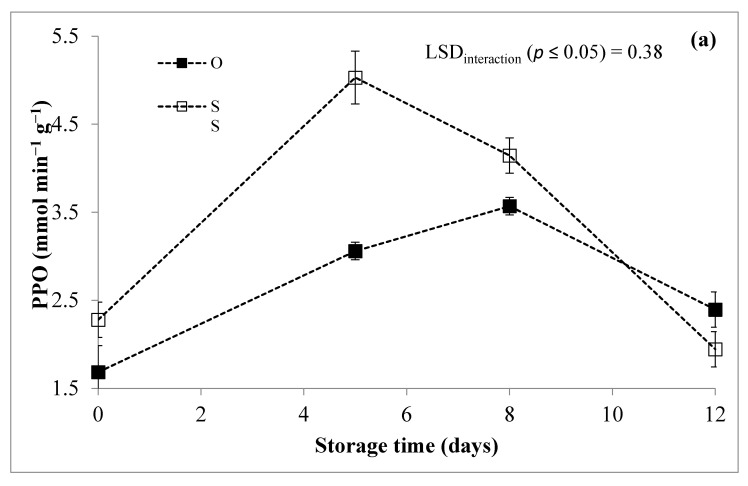

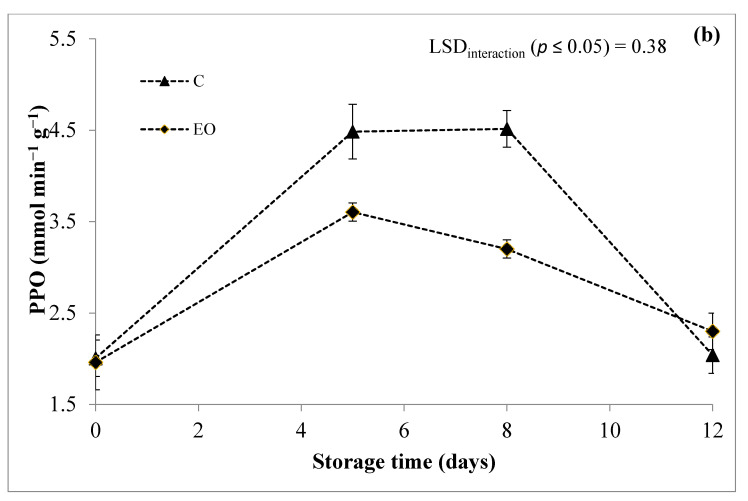
PPO activity of RTC globe artichoke slices as affected by “genotype × storage time” (**a**) and “treatment × storage time” (**b**) interaction. PPO: Polyphenol oxidase; O: “Opera F1”; SS: “Spinoso sardo”.C: Control; EO: Samples packed with essential oil of *F. vulgare.* Data are presented as means ± standard deviation.

Based on the significance of the “G × St” interaction, a similar trend was observed for the PPO in both the selected genotypes (Figure 7a), although the concentration of this enzyme was in the early stages of storage time significantly higher in “Spinoso sardo” compared to “Opera F1”, making the latter genotype potentially the best choice for industrial transformation. The PPO enzyme is activated by stress conditions, resulting after least operations of mincing and cutting and/or by premature ageing phenomena. The genotype “Opera F1” showed at the day of processing (T0), a lower endogenous PPO activity (1.65 mmol min^−1^ g^−1^) respect to “Spinoso sardo” (2.36 mmol min^−1^ g^−1^). In addition, “Spinoso sardo” shows a maximum of activity after the 5th day of storage, while in “Opera F1” it appeared delayed at 8 days, but lower than “Spinoso sardo” in the same sampling time. This tendency should be linked with the browning reactions activated by specific polyphenolic substrates. As previously observed, the extent of the peroxidase (POD, EC 1.11.1.7) isoenzymes in “Opera F1” is really poorer if compared to other globe artichoke genotypes or other crops, such as tomato, melon and strawberry [45]. In addition, variations in polyphenols, PPO and PAL activities were previously reported in cold-stored globe artichoke heads [46]. The subsequent reduction in enzymatic activity may be ascribable to the reduction of the endogenous PPO, which is responsible of the browning phenomena [46], as well as to a molecular rearrangement of chlorogenic acid, 1,3-*O*- and 3,5-*O*-dicaffeoylquinic acids, which are the most representative phenolic compounds in this crop [11,47,48].

Referring to the interaction “T × St” (Figure 7b), both the control (C) and EO treatment had a similar pattern, showing an increase of the values up to the 5th day of refrigerated storage, followed by a gradual decrease at 8 days in EO, confirming the effectiveness of the treatment in reducing the incidence of the polymerization of quinones in brown melanoidins than the control C. It should be noted that the initial browning phenomena could be also attributed to an increased availability of PPO due to its solubilization from the cell wall, mediated mainly by polygalacturonase (EC 3.2.1.15), and pectin methyl esterase (EC 3.1.1.11) [7,10].

### 3.4. Microbiological Quality

**Figure 8:**
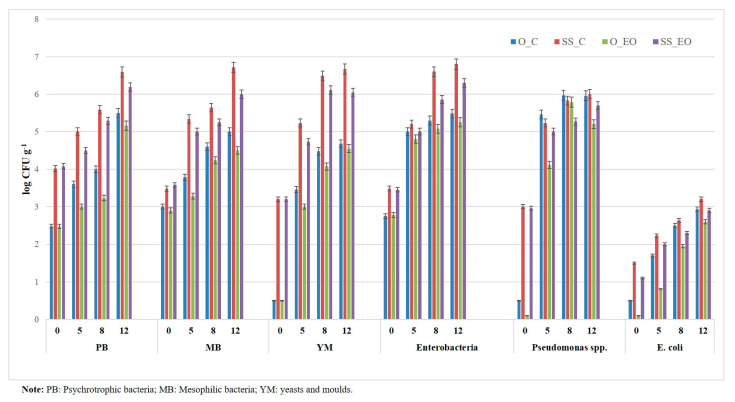
Microbiological parameters of RTC globe artichoke slices as affected by “genotype × post-harvest treatment × storage time” interaction. LSD_interaction_ (*p ≤* 0.05): 0.06 (total mesophilic bacteria, MB); 0.07 (total psychrotrophic bacteria, PB); 0.05 (yeasts and molds, YM); 0.07 (Enterobacteria, TEB); 0.08 (*Pseudomonas* spp.); 0.08 (*E. coli*). Data are presented as means ± standard deviation.

Table 1 shows that each of the three considered main factors (G, T, and St) significantly affected the studied microbiological parameters of RTC globe artichoke slices (*p* ≤ 0.001). Regarding G, higher average microbial counts were recorded for “Spinoso sardo” than “Opera F1” (Table 2), probably for the fact that “Spinoso sardo” is characterized by less assurgent leaves and shorter flowering stems thus exposing heads to a more pronounced microbial contamination from the environment. Referring to St, all microbial groups gradually increased throughout the storage period (Table 2, Figure 8). In particular, in none of the sampling times PB and MB overcame the limit of 8 log CFU g^−1^ suggested by CNERNA-CNRS [49], of 7.7 log CFU g^−1^ imposed by the French law for fresh-cut vegetables [50] and of 7 log CFU g^−1^ as maximum value at expiry date dictated by the Spanish regulation for prepared meals [51]. Instead, the average YM levels at 8 and 12 day (6.03 log CFU g^−1^) moderately exceeded the relative limit of 5 log CFU g^−1^ recommended by CNERNA-CNRS [49], but not causing sensory defects. The average Enterobacteria over the storage period was higher than that reported by Licciardello et al. [9], being influenced by the higher counts evidenced for “Spinoso sardo” already starting from the processing day; however, the average *E. coli* plate count was 2.91 log CFU g^−1^ at the end of the considered period, in conformity with European Regulation EC 1441/2007 [52]. Regarding T, when EO emitter was added to the package system, an average decrease of about 0.50 log CFU g^−1^ was observed for all microbial groups. As reported in Table 2, the “G × T × St” interaction significantly influenced all the considered microorganisms, which were significantly higher in control RTC slices than in those packed with EO emitter (Table 2, Figure 8). The greatest decrements were noticed at 12 d for MB, YM and Enterobacteria in “Spinoso sardo” (0.72, 0.62, and 0.5 log CFU g^−1^, respectively) and for *Pseudomonas* spp. in “Opera F1” samples (0.76 log CFU g^−1^).

### 3.5. Sensory Analysis

The sensory attributes that significantly differentiated, at each sampling time, the RTC globe artichoke slices of the two studied genotypes were shown in Table 3. The intensity (expressed as mean score) was reported only for the significantly different attributes.

At 0, 8, and 12 days, samples were perceived as similar by panelists, except for fennel odor and flavor, while after 5 days of refrigerated storage the samples were significantly different also for bitter attribute. The latter was more intense in the control C as especially observed for “Opera F1”. As expected, the samples packed with EO emitter showed the highest intensities of fennel odor and flavor, regardless of genotype (Table 3).

## 4. Conclusions

The present findings revealed that both globe artichoke genotypes possess a good attitude to be processed as RTC slices, although with a difference response to the applied technological conditions: “Opera F1” showed the best performances for color appearance, thanks to the lower isoenzymes activity, texture and chemical indexes (i.e., TPC, AsAC and AA), while “Spinoso sardo” showed lower ML over the storage time. The addition of *F. vulgare* EO emitter into the packaging system did not influence ML, color appearance and texture, while its presence slowed down the consumption of O_2_, better preserved texture when compared to the control and more effectively controlled PPO activity and antioxidants (AsAC and TPC) retention during the cold storage.

As regards the microbiological quality, all the considered microbial groups gradually increased until the end of the storage period, with a significant higher extent in control RTC globe artichoke slices than in those packed with EO emitter, confirming the inhibiting role played by EO of *F. vulgare*. In addition, the EO emitter did not influence negatively the sensory profile of RTC globe artichoke slices after microwave cooking. Control and active-packaged samples were considered similar by panelists, with the exception of fennel odor and flavor that of course were revealed in samples packed with EO emitter.

Our findings demonstrate the potential application of a *F. vulgare* EO emitter, within an active package system, as a promising technological strategy for preserving quality attributes and reducing microbial spoilage of RTC globe artichoke slices.

## Figures and Tables

**Table 1:** Mean square as absolute value and percentage of total (in brackets) of effects resulting from analysis of variance.

(a)				**Qualitative Trait**									
	**Source of Variation**	**df**	**ML%**	**O_2_%**	**CO_2_%**	**TPC**	**AsAC**	**AA**	**Text**	L*	a*	b*	**∆E**
	Genotype (G)	1	0.13 *** (2.46)	4.20 ^NS^ (3.02)	4.10 ^NS^ (5.84)	43.01 * (4.75)	2485 *** (15.24)	304 *** (38.07)	347.49 * (20.02)	0.03 ^NS^ (0.3)	1.87 ^NS^ (1.43)	0.04 ^NS^ (0)	0.81 ^NS^ (1.4)
	Treatment (T)	1	0.6 *** (11.82)	0.0 ^NS^ (0)	20.11 * (28.66)	118.20 ** (13.05)	1300 *** (7.98)	112 *** (14.10)	89.28 ^NS^ (5.14)	1.19 ** (6.2)	5.74 ^NS^ (4.4)	1.06 ^NS^ (2.3)	2.11 ^NS^ (3.64)
	Storage time (St)	3	3.54 *** (69.75)	80.56 *** (57.95)	30.31 ** (43.2)	698.14 *** (77.10)	11,577 *** (71)	321 *** (40.25)	113.75 ^NS^ (6.55)	7.35 *** (71.78)	104.1 ** (79.79)	37.54 *** (81.38)	45.41 *** (78.36)
	(G) × (T)	1	0.0 ^NS^ (0)	10.89 ^NS^ (7.83)	2.28 ^NS^ (3.25)	1.58 ^NS^ (0.17)	4.5 ^NS^ (0.03)	0.08 ^NS^ (0.01)	10.27 ^NS^ (0.6)	0.49 ^NS^ (4.79)	0.31 ^NS^ (0.24)	0.11 ^NS^ (0.24)	0.77 ^NS^ (1.33)
	(G) × (St)	3	0.33 *** (6.5)	15.90 * (11.44)	3.32 ^NS^ (4.73)	33.23 * (3.67)	118 ^NS^ (0.72)	32 *** (4.04)	333.91 * (19.24)	0.14 ^NS^ (1.3)	7.51 ^NS^ (5.8)	2.88 ^NS^ (6.2)	2.11 * (3.64)
	(T) × (St)	3	0.36 *** (7.09)	16.26 * (11.70)	6.91 ^NS^ (9.85)	6.45 * (0.71)	815 *** (5)	21 *** (2.64)	338.91 * (19.52)	0.09 ^NS^ (0.9)	1.04 ^NS^ (0.8)	0.40 ^NS^ (0.9)	6.12 *** (10.56)
	(G) × (T) × (St)	3	0.12 *** (2.36)	11.21 ^NS^ (8.06)	3.13 ^NS^ (4.46)	4.89 ^NS^ (0.54)	5.2 ^NS^ (0.03)	7 ^NS^ (0.89)	502.20 ** (28.93)	0.95 ** (9.28)	9.89 ^NS^ (7.58)	4.10 ^NS^ (8.9)	0.62 ^NS^ (1.1)
	Total mean square		5.07	139.02	70.16	905.49	16,306	798	1735.81	10.24	130.46	46.13	57.95
(b)				**Qualitative Trait**									
	**Source of Variation**	**df**		**MB**	**PB**		**YM**	**Enterobacteria**		***Pseudomonas***		***E. coli***	**PPO**
	Genotype (G)	1		11.59 *** (55)	17.66 *** (61.71)		33.84 *** (58.5)	4.69 *** (24.41)		4.13 *** (10.31)		2.12 *** (4.35)	4.14 *** (22.73)
	Treatment (T)	1		1.06 *** (5)	1.06 *** (3.7)		0.77 *** (1.3)	0.49 *** (2.55)		1.75 *** (4.37)		7.51 *** (15.42)	1.58 *** (8.68)
	Storage time (St)	3		7.74 *** (36.73)	9.49 *** (33.16)		22.70 *** (39.23)	13.46 *** (70.07)		30.07 *** (75.04)		26.70 *** (54.81)	9.19 *** (50.47)
	(G) × (T)	1		8 × 10^−4 NS^ (0)	0.02 *** (0)		0.03 *** (0)	0.07 *** (0.36)		0.29 *** (0.72)		1.07 *** (2.2)	0.09 ^NS^ (0.5)
	(G) × (St)	3		0.53 *** (2.5)	0.22 *** (0.7)		0.40 *** (0.7)	0.37 *** (1.93)		3.49 *** (8.71)		8.46 *** (17.37)	2.07 *** (11.37)
	(T) × (St)	3		0.12 *** (0.6)	0.13 *** (0.45)		0.09 *** (0.16)	0.10 *** (0.5)		0.16 *** (0.4)		2.22 *** (4.56)	1.12 *** (6.15)
	(G) × (T) × (St)	3		0.03 *** (0.14)	0.04 *** (0.14)		0.03 *** (0)	0.03 *** (0.16)		0.18 *** (0.45)		0.63 *** (1.29)	0.02 ^NS^ (0.1)
	Total mean square			21.07	28.62		57.86	19.21		40.07		48.71	18.21

Note: ML: Mass loss; TPC: Total polyphenol content; AsAC: Ascorbic acid content; AA: Antioxidant activity; Text: Texture (F_max_); color parameters (L*, a*, b*); ∆E: total color difference; df: Degrees of freedom. MB: Mesophilic bacteria; PB: Psychrotrophic bacteria; YM: Yeasts and molds; PPO: Polyphenol oxidase; *, ** and *** indicate significant at *p* ≤ 0.05, *p* ≤ 0.01 and *p* ≤ 0.001, and ^NS^, not significant.

**Table 2:** Qualitative traits of ready-to-cook (RTC) globe artichoke slices as affected by the main factors under study.

(a)	**Main Factor**	**Qualitative Trait**										
		**ML%**	**O_2_%**	**CO_2_%**	**TPC (g kg^−1^ DM)**	**AsAC (mg kg^−1^ DM)**	**AA (% DPPH inhibition)**	**Text (N)**	L*	a*	b*	**∆E**
	Genotype											
	*OPERA F1*	1.62 ^a^	4.57 ^a^	12.26 ^a^	21.26 ^a^	118 ^a^	56.6 ^a^	63.83 ^a^	92.80 ^a^	2.17 ^a^	0.31 ^a^	2.79 ^a^
	*SPINOSO SARDO*	1.49 ^b^	5.29 ^a^	12.98 ^a^	18.94 ^b^	100 ^b^	50.4 ^b^	57.24 ^b^	92.87 ^a^	2.65 ^a^	0.24 ^a^	3.11 ^a^
	Treatment											
	Control	1.42 ^b^	4.94 ^a^	13.41 ^a^	18.18 ^b^	103 ^b^	51.6 ^b^	62.20 ^a^	93.03 ^a^	1.98 ^a^	0.46 ^a^	2.69 ^a^
	Essential oil	1.69 ^a^	4.92 ^a^	11.83 ^b^	22.02 ^a^	116 ^a^	55.4 ^a^	58.86 ^a^	92.65 ^b^	2.83 ^a^	0.09 ^a^	3.21 ^a^
	Storage time (day)											
	0	0.62 ^d^	9.24 ^a^	11.36 ^b^	31.6 ^a^	165 ^a^	61.7 ^a^	64.82 ^a^	93.26 ^b^	3.43 ^a^	−0.53 ^b^	-
	5	1.56 ^c^	2.14 ^c^	14.77 ^a^	23.78 ^b^	102 ^b^	55.3 ^b^	55.80 ^a^	92.03 ^c^	4.47 ^a^	−0.92 ^b^	3.65 ^b^
	8	1.77 ^b^	3.02 ^b,c^	13.77 ^a^	13.15 ^c^	90 ^c^	48.8 ^c^	59.79 ^a^	93.99 ^a^	−2.93 ^b^	3.51 ^a^	5.70 ^a^
	12	2.18 ^a^	5.33 ^b^	10.61 ^b^	11.87 ^c^	80 ^d^	48.2 ^c^	61.71 ^a^	92.07 ^c^	4.68 ^a^	−0.95 ^b^	2.46 ^c^
(b)	**Main Factor**	**Qualitative Trait**										
		**MB**	**PB**	**YM**	**Enterobacteria**		***E. coli***		***Pseudomonas***		**PPO**	
		**(log CFU g^−1^)**									**(mmol min^−1^ g^−1^)**	
	Genotype											
	*OPERA F1*	3.89 ^b^	3.64 ^b^	3.13 ^b^	4.53 ^b^		1.64 ^b^		4.12 ^b^		2.65 ^b^	
	*SPINOSO SARDO*	5.09 ^a^	5.13 ^a^	5.18 ^a^	5.30 ^a^		2.23 ^a^		4.84 ^a^		3.37 ^a^	
	Treatment											
	Control	4.68 ^a^	4.57 ^a^	4.31 ^a^	5.04 ^a^		2.15 ^a^		4.72 ^a^		3.23 ^a^	
	Essential oil	4.31 ^b^	4.20 ^b^	4 ^b^	4.79 ^b^		1.72 ^b^		4.25 ^b^		2.79 ^b^	
	Storage time (day)											
	0	3.21 ^d^	3.23 ^d^	1.8 ^d^	3.07 ^d^		0.8 ^d^		1.63 ^c^		2.01 ^b^	
	5	4.33 ^c^	3.99 ^c^	4.08 ^c^	4.96 ^c^		1.68 ^c^		4.93 ^b^		4.03 ^a^	
	8	4.92 ^b^	4.51 ^b^	5.28 ^b^	5.70 ^b^		2.35 ^b^		5.70 ^a^		3.83 ^a^	
	12	5.52 ^a^	5.82 ^a^	5.46 ^a^	5.92 ^a^		2.91 ^a^		5.68 ^a^		2.16 ^b^	

Note: ML: Mass loss; TPC: Total polyphenol content; AsAC: Ascorbic acid content; AA: Antioxidant activity; Text: Texture (F_max_); color parameters (L*, a*, b*); ∆E: total color difference. MB: Mesophilic bacteria; PB: Psychrotrophic bacteria; YM: Yeasts and molds; PPO: Polyphenol oxidase. Different letters within the same qualitative trait and main factor show significant differences (LSD test, *p* ≤ 0.05).

**Table 3:** Mean scores of the significant sensory attributes of RTC globe artichoke slices as affected by genotype and post-harvest treatment under study.

		Treatment			
Storage Time (d)	Attribute	C		EO	
		“Opera F1”	“Spinoso sardo”	“Opera F1”	“Spinoso sardo”
0	Fennel odor	1.0 ^a^^,^^1^	1.0 ^a^	3.2 ^b^	3.4 ^b^
	Fennel flavor	1.0 ^a^	1.0 ^a^	3.4 ^b^	3.3 ^b^
5	Fennel odor	1.0 ^a^	1.0 ^a^	3.6 ^b^	4.5 ^b^
	Bitter	4.8 ^b^	2.7 ^a^	3.2 ^ab^	2.5 ^a^
	Fennel flavor	1.0 ^a^	1.0 ^a^	3.9 ^b^	4.3 ^b^
8	Fennel odor	1.0 ^a^	1.0 ^a^	2.6 ^b^	4.5 ^c^
	Fennel flavor	1.0 ^a^	1.0 ^a^	3.2 ^b^	5.1 ^c^
12	Fennel odor	1.0 ^a^	1.0 ^a^	3.6 ^b^	3.3 ^b^
	Fennel flavor	1.0 ^a^	1.0 ^a^	3.9 ^b^	3.8 ^b^

^1^ Values marked with different letters in the same row are significantly different (*p ≤* 0.05) according to the LSD test. C: Control; EO: Samples packed with essential oil of *F. vulgare*.

## Data Availability

Main data are contained within the article; further raw data obtained in this study are available on request from the corresponding author.

## Supplementary Materials

The following are available online at https://www.mdpi.com/2304-8158/10/3/517/s1, Figure S1: Experimental work-flow of Ready-to-Cook (RTC) globe artichoke slices.
