# Supplementary figures and images for: Active Packaging-Releasing System with Foeniculum vulgare Essential Oil for the Quality Preservation of Ready-to-Cook (RTC) Globe Artichoke Slices
Source: Foods. 2021 Mar 2;10(3):517. doi: 10.3390/foods10030517 (PMC8001857; doi:10.3390/foods10030517)

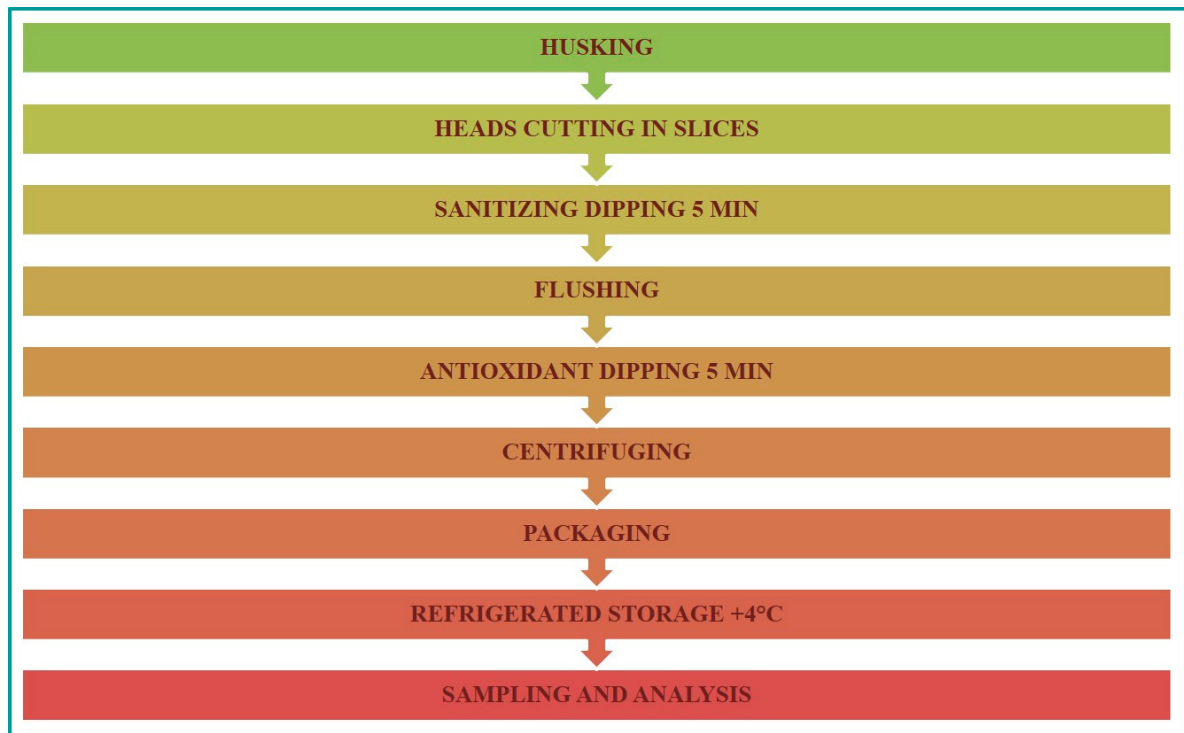

**Figure S1.** Experimental work-flow of Ready-to-Cook (RTC) globe artichoke slices.

Supplement: Supplementary file 1 [file foods-10-00517-s001.pdf]
